# Cryopreservation of *Anopheles stephensi* embryos

**DOI:** 10.1038/s41598-021-04113-x

**Published:** 2022-01-07

**Authors:** Eric R. James, Yingda Wen, James Overby, Kristen Pluchino, Shane McTighe, Stephen Matheny, Abraham Eappen, Stephen L. Hoffman, Peter F. Billingsley

**Affiliations:** 1 Sanaria Inc, Rockville, MD USA; 2 Department of Dermatology, Walter Reed National Military Medical Center, Rockville, MD USA

**Keywords:** Microbiology techniques, Malaria

## Abstract

The ability to cryopreserve mosquitoes would revolutionize work on these vectors of major human infectious diseases by conserving stocks, new isolates, lab-bred strains, and transgenic lines that currently require continuous life cycle maintenance. Efforts over several decades to develop a method for cryopreservation have, until now, been fruitless: we describe here a method for the cryopreservation of *Anopheles stephensi* embryos yielding hatch rates of ~ 25%, stable for > 5 years. Hatched larvae developed into fertile, fecund adults and blood-fed females, produced fully viable second generation eggs, that could be infected with *Plasmodium falciparum* at high intensities. The key components of the cryopreservation method are: embryos at 15–30 min post oviposition, two incubation steps in 100% deuterated methanol at − 7 °C and − 14.5 °C, and rapid cooling. Eggs are recovered by rapid warming with concomitant dilution of cryoprotectant. Eggs of genetically modified *A. stephensi* and of *A. gambiae* were also successfully cryopreserved. This enabling methodology will allow long-term conservation of mosquitoes as well as acceleration of genetic studies and facilitation of mass storage of anopheline mosquitoes for release programs.

## Introduction

Maintenance of *Anopheles* requires continuous culture of the mosquito life cycle, a process that is labor intensive, expensive and vulnerable to colony loss. Until now, no stage of the *Anopheles* life cycle has been preserved; adults can be held for 3–4 weeks^1^ with low survival, while egg survival is limited to 1–10 days depending on humidity and temperature^2,3^.

The goal of this study was to develop a method for cryopreservation of *Anopheles* eggs for the purpose of eventually creating a characterized egg bank under current good manufacturing practice (cGMP) for use in the manufacture of Sanaria®PfSPZ vaccines against malaria^4–9^. The method was also intended to allow banking of new isolates, strains and transgenic lines to aid research on mosquito genetics, malaria and other mosquito-transmitted diseases.

## Materials and methods

### Mosquito embryo collection

*Anopheles stephensi* SDA 500 mosquitoes^10^ have been maintained at Sanaria since 2005 and designated internally as *A. stephensi* SDA500 (9800). Mosquitoes were fed through an artificial membrane on human O + blood (Mid-South Blood Bank, Memphis, TN) by standard methods and housed in 300 × 300 × 300 mm cages^11^. Three days post-feeding, a 150 mm diameter Petri dish (25384-118, VWR) containing ~ 75 mL tap water was introduced into the cage and the mosquitoes were allowed to oviposit for 15 min. Dead mosquitoes or mosquito fragments were removed. The floating eggs were swirled around the Petri dish so the majority adhered to the sides and lid, and the water was decanted from the dish. Eggs were collected using artists paint brushes (No. 4 black-tipped angular shader, MSPCI). Approximately 5,000 eggs could be collected on the upper side of one brush. Surface moisture was removed from the eggs immediately before transfer into cryoprotectant additive (CPA) by blotting the lower side of the brush on an absorbent paper towel.

### Cryoprotectant additives (CPA)

CPAs used were methanol (646377, Sigma-Aldrich) and D4-methanol (321281000, Acros Organics). CPA containers comprised polypropylene 19 mm outer diameter (OD) × 19 mm deep paint mixing pots (10271583, Michaels) housed in 19 mm internal diameter (ID) × 15 mm deep holes milled into a custom aluminium block (see supplementary information, F1) machined to fit onto a Peltier-cooled dual block apparatus (IC22XT, Torrey Pines Scientific). CPA for the first incubation step (2 mL) was equilibrated at − 7 °C for > 1 h prior to addition of eggs. After the first incubation of eggs in CPA at − 7 °C, the container of CPA with eggs was transferred to a second custom aluminium block (see supplementary information, F1) with 3 holes to accept CPA containers similarly supported on a second Peltier-cooled dual block apparatus (IC22XT, Torrey Pines Scientific) at − 14.5 °C. This block also had three 12 mm wide steps running the length of the block on which the rectangular card stock supports were placed at right angles with one end extending beyond the step.

### Sample supports

Card supports for the aliquots of eggs suspended in CPA were cut from 0.14 mm thick black card stock into 15 mm × 5 mm rectangles. These card supports were permeable to CPA and relatively rigid but thin enough to facilitate rapid cooling. The black color of the supports provided contrast for easy visualization of the eggs which are white before melanization begins.

### Samples

The card supports were placed on the steps of the cold block at − 14.5 °C. At the end of the second incubation step at − 14.5 °C, 20 µL aliquots of eggs in CPA were transferred using a P200 pipettor (Ranin) with truncated tip (ID 1.5 mm) to the chilled card supports. Aliquots were allowed to spread on the cards and the CPA to be partially absorbed to reduce surface thickness and improve direct contact of the eggs with the liquid nitrogen (LN_2_) during cooling. Aliquots of eggs were transferred to the card supports 10–30 s before the final cooling step into LN_2_ commenced when the 15 min incubation in CPA at − 14.5 °C was completed.

### Cooling and storage

The refrigerant for cooling was either boiling point LN_2_ at − 196 °C, or chilled LN_2_ between − 202 and − 210 °C that was produced by applying a vacuum source to LN_2_ in a sealed insulated wide mouth vacuum flask (70 mm ID × 110 mm). Each card support with eggs was picked up by its free end using fine forceps and immediately transferred directly into the LN_2_ refrigerant. Each card support was held under the surface of the LN_2_ for ~ 3 s and then transferred to temporary storage in a 6-hole aluminium block (10055-264, VWR) in LN_2_ in an insulated container. Card supports were then transferred into cryovials (Nunc 2.0 mL), previously chilled in LN_2_, and then stored in an isothermal liquid nitrogen vapor phase (LNVP) freezer (V1500AB, Custom Biogenic System).

### Thawing and dilution

To test the larval hatch rates from cryopreserved eggs, sample aliquots on card supports were thawed individually from LN_2_. Thawing and CPA dilution were performed in a single step by rapid transfer of each card support into a stream of water at 23 °C from a 500 mL wash bottle (414004-227, VWR) to a final volume of 10 mL collected into a 50 mm diameter Petri dish (25384-322, VWR). Petri dishes were placed on trays in an incubator (Sanyo) at 28 °C for chorion melanization, embryo development and larval hatching.

### Survival assessment

On day 1 post-thaw, samples in the Petri dishes contained a mix of white non-viable and dark putatively viable eggs. The proportion of dark eggs was used initially to assess the tolerance of eggs to different CPAs (including ethanediol, propanediol, dimethyl sulphoxide, and glycerol), different CPA concentrations, and cooling and warming parameters. *Survival assessment:* Experimental conditions for cryopreservation were developed with melanization as the marker until some eggs developed into embryos with eye spots by days 2–3 post thawing and the first hatched larvae were obtained. Once the first larval hatches had been recorded, the proportion of eggs hatching (number of larvae × 100/number of eggs in sample) on day 3 post thaw was used as the measure of survival. In experiments where full development was assessed, hatched larvae were fed standard larval food (Aqueon Betta fish food and Brewer’s Yeast), pupae were harvested and transferred to bowls in 300 × 300 × 300 mm cages for adult mosquito emergence^11,12^.

### Infectivity assessment

The ability of mosquitoes to be infected with *Plasmodium falciparum* (Pf) was determined by feeding mosquitoes through an artificial membrane on blood cultures containing 0.5% stage V Pf gametocytes^12^.

## Results and discussion

The chitin-rich chorion of anopheline eggs^13^ is a barrier to water movement out of the embryo and to the entry of CPAs, and methods to increase permeability have been the main focus of previous studies aimed at cryopreservation^14–16^. *Drosophila melanogaster* eggs were successfully cryopreserved only after treatment with benzalkonium chloride and heptane to allow permeation of the two CPAs, glycerol and ethanediol, across the egg chorion^17,18^*.* This same treatment was lethal for *Anopheles* eggs: the chorion structure, embryo tegument, and permeability to water and CPAs^19^ are markedly different from *Drosophila*. However, at oviposition, *Anopheles* eggs are soft, non-sclerotized, and more readily water permeable^20^*,* attributes that decline as a function of embryo age.

**Figure 1:**
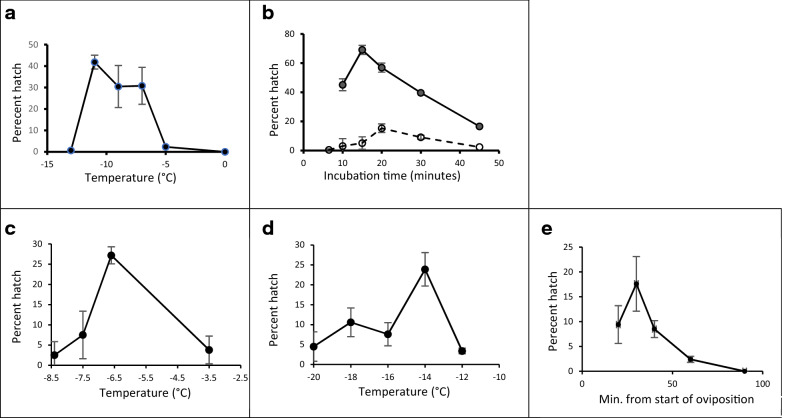
Parameters for oviposition and incubation in cryoprotectant additive (CPA: methanol) for *A. stephensi* egg cryopreservation. (**a**) Survival (percent hatch) of eggs following incubation in 100% methanol for 15 min at temperatures between 0 and − 13 °C without subsequent cooling and with recovery into water at 23 °C. (**b**) Survival (percent hatch) of eggs following incubation in 100% methanol for periods of 7–45 min at − 11 °C and subsequent dilution into water at 23 °C. Eggs directly warmed/diluted from − 11 °C without being rapidly cooled by plunging into LN_2_ (solid line); eggs incubated at − 11 °C and subsequently plunged into LN_2_ before warming/dilution (dashed line). (**c**) Optimization of the incubation temperature for the first CPA incubation step with the second incubation step held constant at − 14.5 °C for 15 min. (**d**) Optimization of the incubation temperature for the second CPA incubation step with the first incubation step held constant at − 7 °C for 6.5 min. (**e**) Effect of oviposition time on survival: eggs were oviposited onto water in Petri dishes for 15 min, harvested and then held an additional 5–75 min before adding to CPA (single incubation at − 11 °C for 15 min). The data points represent the age of the oldest eggs in each sample (20–90 min old).

Methanol, though not well tolerated by many cell types, is a highly effective CPA for a number of organisms, including protists^21,22^*,* trematodes^23,24^*,* nematodes^25^*,* and also fish spermatozoa and eggs^26,27^. Methanol, the lowest molecular weight CPA, appears to be the only CPA that can permeate *Anopheles* eggs, and outside of specific temperatures and with extended exposure times, it is toxic. At − 5 °C few embryos survived a 15-min exposure to 100% methanol and none survived temperatures above − 5 °C (Fig. 1a). Nor did embryos survive a 15 min exposure to methanol at − 13 °C, in this case presumably due to ice formation within the embryos before the methanol could permeate sufficiently or before enough water had been removed. However, *Anopheles* embryos tolerated methanol at temperatures within these bounds with optimal recovery at − 11 °C (Fig. 1a,b for non-cryopreserved eggs). Optimal survival using a single step incubation in methanol at − 11 °C followed by plunge into LN_2_ was obtained after incubating the eggs for 20 min under which conditions 15.3 ± 4.3% of the eggs hatched (Fig. 1b dashed line).

The proportion of larvae that hatched from eggs cryopreserved using a single incubation temperature (Fig. 1b,e) was increased if the incubation in methanol was performed in two steps at two different temperatures (Fig. 1c,d), first at − 7 °C for 6.5–7 min and subsequently at − 14.5 °C for 15 min. With this method, the partial dehydration and partial CPA permeation that occurs during the first incubation step allowed the eggs to survive cooling to below − 13 °C (shown to be lethal in Fig. 1a), making further dehydration and methanol permeation possible during the extended second incubation step at − 14.5 °C. The toxicity of methanol is both temperature and time dependent, and the time that the eggs can tolerate immersion in methanol is extended by the lower temperature of the second incubation step.

The effect of the two incubation steps is to maximize the reduction in the amount of freezable intracellular water both through CPA-induced dehydration and by increasing the internal CPA concentration while minimizing CPA toxicity. These processes will combine to increase the viscosity of the intracellular contents enhancing their propensity to vitrify during rapid cooling in LN_2_. The results of experiments conducted to optimize the temperatures for the two incubation steps are shown in Fig. 1c,d.

At the end of the second incubation step, the eggs dispensed in 20 µL aliquots on card supports and held at − 14.5 °C, were plunged directly into boiling point LN_2_ (cooling rate of − 12,240 ± 151 °C min^−1^) or into LN_2_ chilled to − 202 °C or below (cooling rate − 87,864 ± 6315 °C min^−1^).

In twelve experiments comparing boiling point vs chilled LN_2_, hatch rates were marginally, but not statistically significantly, higher for samples cooled using chilled LN_2_. Similarly, a small, but not statistically significant increase in hatch rate was obtained using D4-deuterated methanol which has an approximately 10.7% higher viscosity than H4-methanol (at 25 °C)^28^.

Following storage in LNVP below − 150 °C, recovery was accomplished through simultaneous thawing and dilution to remove CPA by holding the card support with eggs under a stream of water directed into a Petri dish. This operation was performed rapidly. The optimal water temperature was 23 °C (data not shown) which produced a warming rate of ~ 30,400 °C min^−1^ and a 1:500 dilution of the methanol.

Embryos of most species cryopreserve optimally when cells are undifferentiated^29,30^*,* when the embryos are potentially able to tolerate the loss of some proportion of their cells without subsequent development being impaired. Counterintuitively, successful cryopreservation of *Drosophila* eggs was optimal at 12–15 h post oviposition^17,18^ when embryo development is nearly complete. *Anopheles* eggs are fertilized at oviposition, and the embryo develops as a syncytium until cell wall involution which becomes visible at approximately 4–6 h^16^*. A. stephensi* eggs require 48–72 h at 28 °C for larval emergence. By harvesting *Anopheles* eggs soon after oviposition when the eggshell is soft^16,19^ and relatively porous to water and methanol, there is no need for permeabilization. Maximum survival of eggs exposed to 100% methanol at − 11 °C for 15 min and cooled to − 196 °C occurred at 15–30 min after oviposition (Fig. 1e). The subsequent decline may be related to the progressive chemical hardening of the chorion affecting permeability and/or to changes in the developing embryo, including the early stages of cellular differentiation.

After storage in LNVP for 24 and 60 months, hatch rates were 28.2 ± 5.5% and 23.3 ± 2.0%, respectively. Cryopreserved eggs thawed and subsequently maintained at 28 °C in water hatched on days 2 and 3, the same timing as control (not cryopreserved) eggs, and the development time of larvae through to pupation followed a similar timeline for cryopreserved and control eggs. The proportion of hatched larvae derived from cryopreserved eggs that subsequently pupated was 76.3%, and of these pupae 87% yielded adult mosquitoes of which 49.7% were females.

The feeding behavior of adult mosquitoes derived from cryopreserved eggs was unaffected by cryopreservation (Table 1). The rate of development of mosquitoes obtained from cryopreserved eggs fed on *Plasmodium falciparum* (Pf)*-*infected blood meals was similar to that of mosquitoes routinely infected at our facility; the prevalence of infection (50%) was lower but infection intensities were comparable to our routine infections. Adult female mosquitoes derived from cryopreserved eggs were fed on uninfected blood and a second generation of females then fed on Pf-infected blood—the prevalence of Pf infection in this case reached 93%. This was not intended to be a controlled experiment: the purpose was to determine whether different generations of mosquitoes reared from cryopreserved eggs could still be infected with *Plasmodium falciparum.*

**Table 1:** *P. falciparum* infection of adult *A. stephensi* derived from cryopreserved eggs.

	First generation	Second generation
Number of mosquitoes dissected	6	30
Prevalence of PfSPZ infection (percent mosquitoes infected)	50%	93%
Intensity of infection (mean PfSPZ/mosquito)	30,833	57,835
Mean PfSPZ per infected mosquito	61,667	62,188
Range of PfSPZ per infected mosquito	49,000–78,000	250–276,500

Adult mosquitoes grown from cryopreserved eggs and second-generation adults reared from eggs laid by those first generation cryopreserved mosquitoes were fed on cultured stage V Pf gametocytes, and the numbers of sporozoites (PfSPZ) in the salivary glands were determined 14 days later.

We present here, a method that reproducibly yields ~ 25% hatched first instar larvae of *A. stephensi* mosquitoes from cryopreserved eggs. The critical components of the technique are the time of harvesting the eggs after laying (15–30 min), the use of absolute (24.8 M) methanol as the CPA, the two-step exposure to CPA (− 7 °C for 6.5–7 min followed by − 14.5 °C for ~ 15 min), rapid cooling into LN_2_ (> − 12,240 °C min^−1^), and rapid thawing (+ 30,000°Cmin^−1^) with CPA dilution (1:500 in water at 23 °C). The method is improved marginally by using D4-methanol and chilled LN_2_. No pretreatment to permeabilize the egg chorion is required, although surface water is removed before adding the eggs to CPA. To date this method has been successful with all strains of *A. stephensi* tested^11,12^ including a fragile LRIM1 knock out transgenic line^30,31^ and also with *A. gambiae* (Keele) in one trial experiment (data not shown). We anticipate that the method should work with newly colonized strains.

## Supplementary Information

Supplementary Information.
