# Supplementary material for: Cryopreservation of Anopheles stephensi embryos
Source: Sci Rep. 2022 Jan 7;12:43. doi: 10.1038/s41598-021-04113-x (PMC8741979; doi:10.1038/s41598-021-04113-x)
Supplement: Supplementary file 1 — Supplementary Information. [file 41598_2021_4113_MOESM1_ESM.pdf]

Supplementary Figure 1

Cryopreservation of *Anopheles stephensi* embryos

Eric R. James, Yingda Wen, James Overby, Kristen Pluchino, Shane McTighe, Stephen Matheny, Abraham Eappen, Stephen L. Hoffman, Peter F. Billingsley

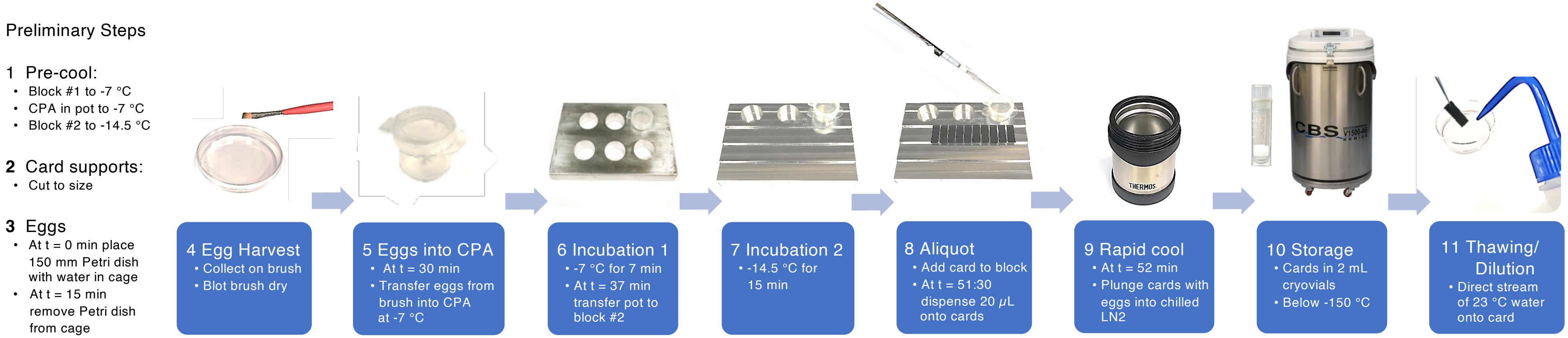

Figure Title: Supplementary Figure 1.

Figure legend: Diagram outlining the steps in the methodology for cryopreserving eggs of *Anopheles* mosquitoes.
